# Assessing the Role of Livestock in Big Cat Prey Choice Using Spatiotemporal Availability Patterns

**DOI:** 10.1371/journal.pone.0153439

**Published:** 2016-04-11

**Authors:** Arash Ghoddousi, Mahmood Soofi, Amirhossein Kh. Hamidi, Tanja Lumetsberger, Lukas Egli, Igor Khorozyan, Bahram H. Kiabi, Matthias Waltert

**Affiliations:** 1 Workgroup on Endangered Species, J.F. Blumenbach Institute of Zoology and Anthropology, Georg-August-Universität Göttingen, Göttingen, Germany; 2 Persian Wildlife Heritage Foundation, Tehran, Iran; 3 Faculty of Biological Sciences, Shahid Beheshti University, G.C., Tehran, Iran; U.S. Geological Survey, UNITED STATES

## Abstract

Livestock is represented in big cat diets throughout the world. Husbandry approaches aim to reduce depredation, which may influence patterns of prey choice, but whether felids have a preference for livestock or not often remains unclear as most studies ignore livestock availability. We assessed prey choice of the endangered Persian leopard (*Panthera pardus saxicolor*) in Golestan National Park, Iran, where conflict over livestock depredation occurs. We analyzed leopard diet (77 scats) and assessed wild and domestic prey abundance by line transect sampling (186 km), camera-trapping (2777 camera days), double-observer point-counts (64 scans) and questionnaire surveys (136 respondents). Based on interviews with 18 shepherds, we estimated monthly grazing time outside six villages with 96 conflict cases to obtain a small livestock (domestic sheep and goat) availability coefficient. Using this coefficient, which ranged between 0.40 and 0.63 for different villages, we estimated the numbers of sheep and goats available to leopard depredation. Leopard diet consisted mainly of wild boar (*Sus scrofa*) (50.2% biomass consumed), but bezoar goat (*Capra aegagrus*) was the most preferred prey species (I_j_ = 0.73), whereas sheep and goats were avoided (I_j_ = -0.54). When absolute sheep and goat numbers (~11250) were used instead of the corrected ones (~6392), avoidance of small livestock appeared to be even stronger (I_j_ = -0.71). We suggest that future assessments of livestock choice by felids should incorporate such case-specific corrections for spatiotemporal patterns of availability, which may vary with husbandry methods. Such an approach increases our understanding of human-felid conflict dynamics and the role of livestock in felid diets.

## Introduction

As part of the wide diet spectrum of felids, livestock has been depredated throughout the world [1]. Livestock depredation causes serious damage to local economies and creates or reinforces negative attitudes toward conservation initiatives and felids [1]. Moreover, conflicts may result in the application of lethal control of felids of high conservation value [1]. Despite different efforts of husbandry (e.g. night corrals, shepherds and guarding dogs) practiced to minimize losses, livestock still constitutes a considerable proportion to felid diets in some parts of the world [2–4]. Therefore, understanding the livestock choice by felids in the world constantly changing due to anthropogenic modifications is of high importance for conservation [1, 5]. Prey choice is a complex trophic relationship defined as the disproportional use of a resource given its availability, relative to all other resources with factors such as prey abundance, body mass, group size and injury threat, as well as habitat features playing important roles [5–6]. Decline of natural prey abundance, lax husbandry methods, and individual predator behavior have all been suggested as the important factors in felid depredation on livestock [3, 7–10].

Due to the domestication process, livestock may have lost their agility to predator attacks and are easier targets for predators compared to wild prey [7]. However, humans provide livestock with different means of protection, potentially minimizing the predator’s chance for depredation and threatening predators themselves [11]. Nevertheless, preference or avoidance of livestock in felid diet in comparison with wild prey has been rarely quantified [12–14]. The exclusion of livestock from most felid prey choice analyses may originate from the lack of information on their numbers or husbandry practices [15]. As livestock predation risks may vary depending on different husbandry methods [2–4, 11–13], absolute livestock numbers may be inadequate to assess the role of livestock in felid prey choice [15]. Therefore, we argue that using spatiotemporal livestock availability stemming from different husbandry methods may result in meaningful incorporation of livestock into prey choice studies. Such spatiotemporal corrections may provide more realistic information on preference of domestic prey by felids and the efficiency of husbandry methods. In this study, for the first time to our knowledge, we incorporate the spatiotemporal livestock availability into a felid prey choice study to better understand the dynamics of a human-felid conflict.

The leopard (*Panthera pardus*) is known as a predator preying on at least 111 wild species throughout its range [16]. This diversity of prey species and the habitats they inhabit shows the flexibility of this species in tolerating different conditions [16]. However, leopards are known to prefer preying on medium-sized ungulates within a weight range of 1–45 kg, which occur in small herds, pose a minimal risk of injury and live in habitats with moderate cover for hunting [16–19]. Leopards are also responsible for livestock depredation in much of their range, but their preference/avoidance of livestock is unknown [2, 20–21].

We assessed the prey choice of the Persian leopard (*P*. *pardus saxicolor*) in Golestan National Park (GNP), Iran, where the conflict with humans is of great concern for the conservation of this globally endangered subspecies [22]. We used scat sampling for dietary analysis and wild and domestic prey population assessment by means of distance sampling, camera trapping, point-counts and questionnaire surveys. Using a livestock availability coefficient based on the spatiotemporal patterns of livestock grazing outside conflict villages, we aimed to assess the availability of livestock vs. wild prey in a more informed way than by using crude livestock numbers. This application may assist conservation managers by providing a better understanding of big cat preferences of wild and domestic prey and by improving the effectiveness of husbandry methods in mitigating conflicts.

## Material and Methods

### Study area

**Fig 1:**
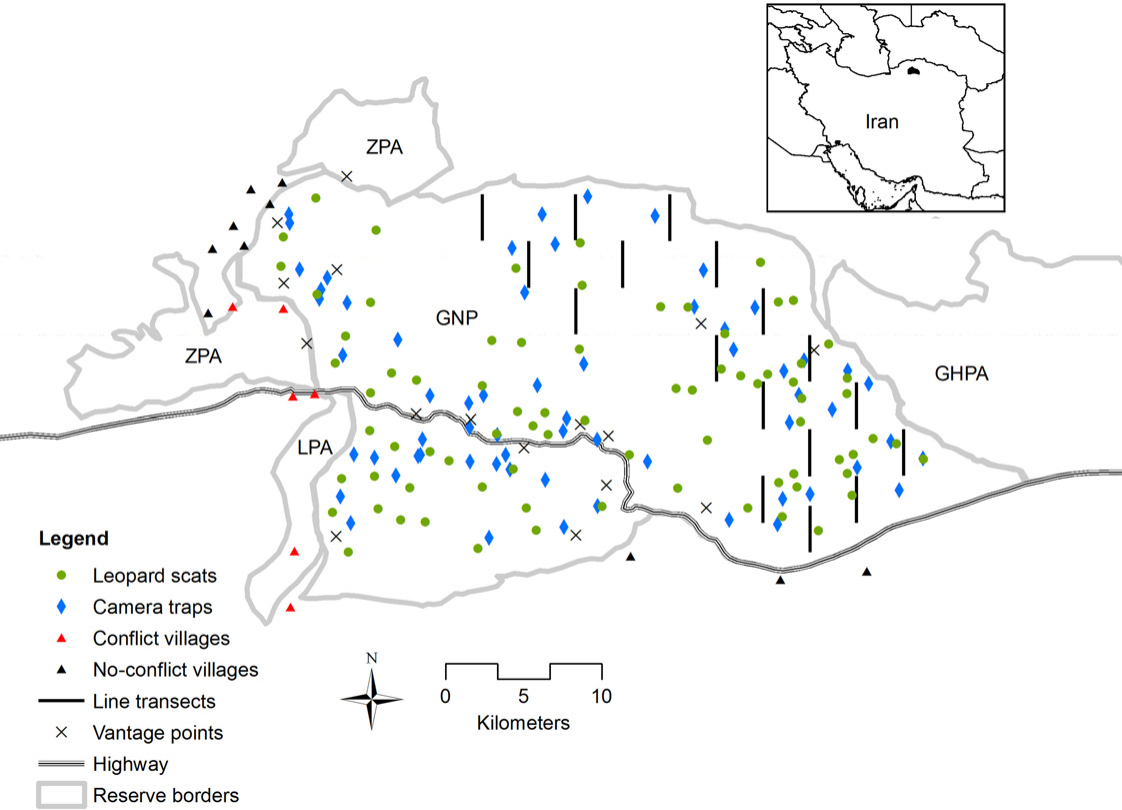
Map of Golestan National Park and the location of scat samples, line transects, camera trap stations and vantage points, as well as the conflict and no-conflict villages and neighboring reserves (LPA: Loveh Protected Area; ZPA: Zav Protected Area; GHPA: Ghorkhod Protected Area).

GNP is located in northeastern Iran from 37°16’43”N and 37°31’35”N to 55°43’25”E to 56°17’48”E (Fig 1), with an area of 874 km^2^. GNP elevation ranges from 450 to 2411 m a.s.l. [23]. The dissimilar mean annual precipitation of 142 and 866 mm in the east and west, respectively, results in varied vegetation types from deciduous forest to steppe and semi-desert [23]. GNP is known to hold the largest global population of the endangered Persian leopard (27 indiv., 95% confidence interval CI = 23–42) and to be among the richest Iranian reserves in ungulates (six species) [24]. However, extensive poaching in GNP due to insufficient acceptance of conservation laws among local communities and lack of appropriate enforcement measures has caused a drastic decline in the ungulate populations in recent decades [24–25]. Around 8815 inhabitants live in 16 villages within less than 2.5 km distance from GNP boundaries (Fig 1) [22]. No villages exist inside GNP boundaries. The main occupation of people in these villages is crop cultivation and livestock rearing, with frequent but illegal grazing of livestock inside the park [22]. When grazing in pastures outside villages, domestic goat (*Capra aegagrus hircus*) and sheep (*Ovis aries*) herds are mostly guarded by shepherds and dogs (*Canis familiaris*). Otherwise, they are kept in pens inside the villages. As domestic goats and sheep graze in mixed herds in the region, we hereafter consider them as a single prey species available to leopard predation and refer to them as ‘small livestock’ [22]. Although domestic sheep and goats may have different movement patterns and their predation risk can be different, such variations are unknown to us and were considered as constant. High quality cattle (*Bos taurus*) are kept in pens and other cattle roam freely in pastures and forests outside villages, normally without protection [22]. Depredation of livestock by leopards occurs in villages around GNP and in many cases, local people do not tolerate such losses and may illegally kill leopards by poisoning the remaining livestock carcasses or shooting [26].

### Leopard diet analysis

We used scat analysis for identification of leopard diet in GNP [27]. Leopards defecate several times from one predation event and use their scats as a territorial sign, which provides valuable dietary information [27]. Faecal sampling of leopards provides comparable results to inspection of kill sites from GPS-collared animals, while the latter approach is more costly and may under-represent small species [4, 28]. As leopards have been recorded all around GNP [24], we collected their scats on an irregular basis throughout the park alongside the main trails or near scrapes [13]. To avoid autocorrelation, we avoided collection of multiple scats from the same location likely originating from the same predation event. Also, we distributed our scat sampling surveys as widely as possible, so that our diet analysis would represent all leopards of GNP (Fig 1). Moreover, leopard diet did not differ between wet and dry seasons, therefore the temporal variation in diet was not considered [29]. The team members (AG, AKH & MS) and park rangers collected leopard scats between 2011 and 2014 and distinguished them from other carnivore scats by their diameter (2–4 cm; mean = 2.85 cm; standard deviation SD = 0.51), cylindrical shape and segmentation into several lobes with pointed ends [29]. The park holds no other big cats after extinction of tiger (*P*. *tigris*) in 1953 [26]; hence, we are confident that our scat sampling was restricted to leopards. The scats were air-dried after collection and stored separately in labeled paper bags at room temperature. The undigested prey remains were cleaned with 70% ethanol and rinsed with distilled water [27]. Ten random hairs were examined macroscopically and microscopically from each scat sample and identified using the previously compiled hair reference catalogue from the study area [30] and other references [31–32]. Frequency of occurrence (*FO*) and percent of occurrence (*PO*) of prey remains in scats were calculated from the number of scats containing a certain *i*-th prey species (*n*_*i*_) by using equations [27]:

$${FO}_{i}\left(\%\right)=(n_{i}/N_{scat})\times 100$$

$${PO}_{i}\left(\%\right)=(n_{i}/N_{item})\times 100$$

To account for more than one prey species per scat, a corrected *FO* (*CFO*) was obtained by allocating equal fractions of the scat to the prey species [33]. To overcome the overrepresentation of smaller prey items in the diet, we used a non-linear biomass correction factor for calculation of prey biomass consumed by leopards [34]:

$${CF}_{1;i}=2.358\left(1-exp(-0.075x)\right)$$

where *CF*_*1*_ is the biomass of the *i*-th consumed prey species per scat against the average body mass of the species *x* (kg). For estimation of prey numbers consumed by leopards, we used another non-linear correction factor:

$${CF}_{2;i}=3.094exp\;\left(-0.5{\left(\left(ln(x/16.370)\right)/2.584\right)}^{2}\right)$$

where *CF*_*2*_ is the number of the *i*-th consumed prey species per scat against the average body mass of the species *x* (kg). Average body mass of prey species was extracted from the literature as 3/4 of average female body mass to account for predation on juvenile and sub-adult individuals [30, 35]. To check whether our scat samples were sufficient to accurately portray leopard diet diversity in GNP, the accumulation curve was computed in EstimateS 9.1 based on the Shannon diversity index [36]. Possible changes in leopard diet profile with higher sample size were assessed by comparing our scat data (n = 77) with the pooled data from this study and an earlier research in GNP (n = 115) using similar methodology [29]. Differences in the prey species *CFOs* between the pooled and original data were tested using the two-way *Z*-test.

### Prey population estimation

We conducted prey population surveys of the top four prey species: wild boar (*Sus scrofa*), bezoar goat (*C*. *aegagrus*), urial *(O*. *vignei*) and small livestock, which contributed over 85% of *PO* in leopard diet (see above). There was insufficient data on abundance of other potential prey species, which occur seldom in leopard diet in our study area. Because of variations in detection probability of prey due to different habitat structure or species ecology, we applied different population estimation methods for each species (see below). We used a stratified random sampling approach for estimation of wild prey abundance.

As there is no reported seasonal migration of wild prey to or from GNP, temporal availability of wild prey was considered as constant. There is no significant difference in the overall diet of leopards between steppe and forest parts of the park [29] and the ranging pattern of leopards in GNP is not restricted to a specific habitat [24]. Therefore, variations in spatial availability of wild prey were not considered as well. However, we acknowledge that there are subtle variations in spatiotemporal availability of wild prey within ranges of different leopard individuals, which we were unable to measure. On the other hand, as domestic prey is available to leopard predation only when grazing outside villages and conflict cases are spatially explicit [37], we considered the spatiotemporal availability of livestock (see below).

#### Line transects

We applied Distance sampling using line transects to estimate the density of urial in GNP [38]. A detailed description of the urial line transect sampling design and modeling is provided elsewhere [25]. We sampled a 340 km^2^ steppe area of the park as the main urial habitat by surveying 17 transects (Fig 1). We used Distance 6.0 software [39] for estimation of urial abundance.

#### Camera trapping

We used the random encounter model (REM) for estimation of wild boar abundance [40], using camera-trapping data from January to December 2011 provided by the Persian Wildlife Heritage Foundation [24]. As we attempted to set up camera traps with a minimum distance of 2 km apart in areas favored by leopards across the park (i.e. wherever leopard signs such as scrapes or scats were detected), we assumed that these locations are random to wild boar movements [41]. Our camera trapping procedure has been described in the literature before [24]. Estimation of wild boar density using the REM method incorporates the number of independent photographic events of the species (*y*), total camera trapping effort (*t*), average daily movement of the species when active (*v*) and average group size (*g*), as well as camera trap-related parameters such as detection distance (*r*) and angle (*θ*) using the following equation [40]:

$$D=\frac{y}{t}\frac{\pi }{vr(2+\theta )}\times g$$

We retrieved *r* and *θ* values from the published literature [40] that used the same brand of camera traps (Deercam DC300; Non Typical Inc., Wisconsin, USA) at 12 m and 0.175 radians, respectively. Since wild boars are considered large animals (50–300 kg in GNP) [35], there is little difficulty in their detection by camera traps and classical approaches in estimation of camera trap parameters seem sufficient [42]. Daily movement of wild boars (*v*) was extracted from a radio-tracking study in a primeval temperate forest, which is ecologically comparable to GNP, as 6.8 ± standard error (SE) 0.57 km.day^-1^ [43]. Average group size of wild boars (*g*) was calculated through the encounters of this species on line transects in GNP (S1 File). We estimated the variance of density using the delta method, as the squared variance of each independently estimated REM parameter added to the squared variance of bootstrapped wild boar encounter rate (*y*/*t*) [40]. We conducted bootstrapping by resampling camera locations 10,000 times with replacement [40].

#### Double observer point-count

To assess the abundance of bezoar goats, which inhabit hardly accessible rocky terrain, we applied a double-observer point-count approach based on mark-recapture theory [44–45]. We followed habitat descriptions in the literature to identify the rugged habitat of bezoar goats in GNP [46–47]. Namely, we used a threshold of 0.03 for the ruggedness index [48] and 40° for slope using the digital elevation model (DEM) in ArcGIS 10.1 (ESRI, Redlands, CA). Moreover, we added a buffer of 200 m as bezoar goats graze in areas near cliffs as well [47]. We excluded isolated habitat patches of less than 3.5 km^2^, where we did not expect any animals to exist. Thereby, in the remaining 53.6 km^2^ of bezoar goat habitat we selected 20 random sampling points with a minimum distance of 3 km (Fig 1). We selected vantage points at 200–500 m away from the hardly accessible bezoar goat habitat using the viewshed function in ArcGIS 10.1 for scanning the sampling points. Each vantage point was visited once by two trained observers (usually one park ranger and one team member: AG, AKH, LE or MS), each equipped with a rangefinder, binoculars and compass. Each observer conducted four alternating independent scans of 15 minutes and recorded sightings up to a maximum distance of 1000 m to equalize detection changes for both observers [44]. For each sighting, observers mapped the location and recorded the number of animals (cluster size) and the distance to the center of the cluster. After finishing the survey, both observers compared their data sheets to identify ‘captures’ (clusters detected by one observer) and ‘recaptures’ (clusters detected by both observers). Due to the relatively low density of the target species, it was unproblematic to distinguish groups and avoid double counting. Point counts were conducted during six fieldwork days (17–19 November and 2–4 December 2014). Due to bad weather conditions and difficult accessibility, we omitted surveying four points. We used the program DOBSERV [49] to model detection probability of bezoar goat clusters based on two capture-recapture models: equal or unequal detection probability between the observers. Methodological details underlying the program are described elsewhere [49]. We used the Akaike information criterion corrected for small sample sizes (AIC_c_) to find the most parsimonious model(s) and selected the best models as those having Δ AIC_c_ < 2 [50]. To translate this number into density, we multiplied it by the average group size and divided by the sampling area. The sampling area was calculated from the overlap of areas visible from vantage points and the identified bezoar goat habitat using the viewshed function in ArcGIS 10.1. The density was then extrapolated to the total bezoar goat habitat to calculate the abundance.

#### Interview survey

We conducted structured questionnaire surveys in March and May 2013 among 136 council members and village heads from 34 villages within the GNP watershed to obtain data on small livestock depredation by leopards [22]. Although leopard depredation on small livestock has been reported in 12 out of 34 studied villages, we considered only villages < 2.5 km away from GNP boundaries (spatial availability). This was done to include only villages with the highest likelihood of attacks by leopards from GNP, according to the earlier results on Persian leopard movements [51] and the highest intensity of conflicts in the vicinity of reserves [21–22, 52]. Details on our interview survey procedure have been provided elsewhere [22]. As spatial characteristics of carnivore attacks on livestock may play an important role in the conflict [4, 21, 53], we used only small livestock data from villages with confirmed leopard attacks in our analysis (spatial availability). In GNP, small livestock are available to leopard predation only during free grazing or corralling at night in the pastures and are not killed while in pens inside villages [2–3, 22, 52]. Having interviewed 18 shepherds, we developed a small livestock availability coefficient (*C*) as the proportion of the average number of grazing days to the number of all days in a month (temporal availability). So, *C* ranged from 0 to 1 per month, where 0 means that small livestock do not graze outside and stay only in village pens and 1 means staying overnight in the fields away from villages all month. When small livestock were grazed during the day and returned to village pens in the evening, we considered *C* as 0.5. Livestock numbers in each conflict village were corrected by multiplying by their corresponding value of *C*. We acknowledge that this is a simplification of predation risk, which can be affected by other husbandry factors as well [11]. However, as small livestock are within the leopard’s preferred prey body mass range [19], cause no injury threat to leopards, and graze mostly inside GNP or its surroundings, we believe their predation probability is determined by availability [3, 52]. Cattle predation by leopards also occurs in the villages around GNP [22]. However, as cattle grazing outside pens and consequently predation by leopard depends on cattle age and breed [2, 13, 22], we were unable to incorporate its availability and excluded cattle from this study.

### Leopard prey preferences

We used Jacob’s index to indicate leopard prey preference *I*_*j*_ [16, 53]:

$$I_{j}=\frac{r_{i}-p_{i}}{r_{i}+p_{i}-2r_{i}p_{i}}$$

where *r*_*i*_ is the proportion of the number of individuals of the *i*-th consumed prey species to all consumed individuals and *p*_*i*_ is the proportion of the abundance of the *i*-th species to the abundance of all prey species. We estimated the numbers of prey individuals consumed by using *CF*_*2*_. Also, we used abundance estimates of wild prey and small livestock (using *C*) for calculation of *p*_*i*_. In calculations of *r*_*i*_ and *p*_*i*_, we used only top four prey species described above and corrected the proportion of prey individuals consumed accordingly. The index ranges from -1 to +1, where +1 indicates maximum preference and -1 indicates maximum avoidance. We considered the prey species as preferred if their Jacob’s index was >0.3 and as avoided if it was <-0.3, with the index between these values indicating predation based on abundance [19]. Differences in leopard prey preference with and without small livestock as a prey species were tested using paired *t*-test. To assess the influence of uncertainty of prey abundance estimates on leopard prey preferences, we ran sensitivity analysis using all different combinations of the 95% CI limits of the abundance of wild prey and small livestock (corrected and uncorrected estimates). We compared the results of sensitivity analysis with the original prey preference. Sensitivity analysis was conducted using R statistical software 3.2.2 (R Foundation for Statistical Computing, 2015).

### Ethics statement

The field surveys including collection of leopard scats, use of camera traps and implementation of ungulate surveys and interviews with local communities in GNP (a state-governed national park) were made possible due to the written approval of the Iranian Department of Environment (DoE) and Golestan provincial office of DoE. Persian Wildlife Heritage Foundation conducted the camera trapping survey [24], with permits obtained from Golestan provincial office of DoE, and granted the use of its data in this research. The interviews in villages surrounding GNP were conducted within a project approved by the DoE and Golestan provincial office of DoE [22]. The interviewees gave their verbal consent as getting written consent could have changed the participants’ perceptions of the purpose of this research and consequently could affect the data quality. By filling up the questionnaire forms, one per participant, the participants gave their consent to take part in this study. Interviewees were informed about the purpose of this study and ascertained about the anonymity and security of their data. The interview survey was in accordance to the ethical guidance of Georg-August-Universität Göttingen. The review boards of DoE and Golestan provincial office of DoE approved the interview procedure of this study. No animal handling was conducted in this research.

## Results

### Leopard diet analysis

In total, we collected 77 scats containing 12 different prey species and one scat of unknown remains; therefore, we used 76 scats in dietary analysis (Table 1). The majority of leopard diet in GNP consisted of wild boar (*FO* = 50.0%; *PO* = 46.3%), followed by bezoar goat (*FO* = 14.5%; *PO* = 13.4%), urial (*FO* = 11.8%; *PO* = 13.4%) and small livestock (*FO* = 13.2%; *PO* = 12.2%). The accumulation curve of the Shannon diversity index leveled-off at approximately 20 samples and reached an asymptote at approximately 50–60 samples, indicating that 76 samples were enough to portray the prey diversity in diet (S1 Fig). A comparison of our sample with the pooled sample (n = 191; S1 Table) did not reveal a significant difference in the *CFO* of the top four prey species (*P*>0.05; *Z*_wild boar_ = 0.46; *Z*_bezoar goat_ = 0.93; *Z*_urial_ = -1.08; *Z*_livestock_ = 0.71). Therefore, we are confident that our sample size was sufficient and reliable to show the diversity and the role of each prey species in leopard diet in GNP. We used the leopard diet data only from our study in the prey preference analyses.

**Table 1:** Results of Persian leopard diet analysis in Golestan National Park, Iran.

Species	Body mass^a^	No. in leopard scats	*CFO*^d^	Biomass consumed^e^		Individuals consumed^f^	
	(kg)	(*n*)	(%)	(kg)	(%)	(*n*)	(%)
**Wild boar *Sus scrofa***	71.5^b^	38	47.8	83.9	50.2	13.6	50.0
**Bezoar goat *Capra aegagrus***	36^c^	11	14.7	24.2	14.5	3.7	13.7
**Urial *Ovis vignei***	34^c^	9	10.7	17.4	10.4	2.7	9.9
**Domestic goat *C*. *a*. *hircus***	44.8^c^	5	6.0	10.2	6.1	1.6	5.8
**Domestic sheep *Ovis aries***	57.1^c^	5	6.7	11.6	7.0	1.8	6.7
**Roe deer *Capreolus capreolus***	20^c^	4	5.4	7.3	4.4	1.3	4.8
**Indian crested porcupine *Hystrix indica***	11^c^	3	3.4	3.3	2.0	0.8	3.0
**Domestic dog *Canis familiaris***	32.2^c^	2	2.7	4.3	2.6	0.7	2.5
**Domestic cattle *Bos taurus***	250^c^	1	1.3	2.4	1.4	0.6	2.1
**Red deer *Cervus elaphus***	98.8^c^	1	1.3	2.4	1.4	0.4	1.5
**Rodents**	-	1	-	-	-	-	-
**Birds**	-	1	-	-	-	-	-
**Unknown**	-	1	-	-	-	-	-
**Total**	-	82	100	167.0	100	~27	100

^a^ 3/4 of mean adult female body mass

^b^ [35]

^c^ from various references cited in [30]

^d^ corrected frequency of occurrence

^e^ based on *CF*_1;*i*_ = 2.358(1 − *exp*(−0.075*x*)) [34]

^f^ based on *CF*_2;*i*_ = 3.094*exp* (−0.5((*ln*(*x*/16.370))/2.584)^2^) [34]

### Prey population estimation

#### Urial

With the total survey effort of 186 km, 1981 urials in 70 clusters were detected. The best-fitting detection function derived from a half-normal key resulted into an estimated density of 12.6 indiv./km^2^ (coefficient of variation CV = 35.5%; 95% CI = 6.2–25.4). The urial population was estimated as 4275 individuals (95% CI = 2117–8632).

#### Wild boar

A total of 2777 trap-nights resulted in 386 wild boar photos. In total, 38 observed groups of wild boar on line transects yielded an average group size of 3.10 ± SE 0.85 individuals. Wild boar density was estimated as 7.4 indiv./km^2^ (CV = 27.0%; 95% CI = 3.5–11.3) and the population size as 6478 individuals (95% CI = 3050–9906).

#### Bezoar goat

We observed 39 bezoar goats in seven clusters during 64 scans of the surveyed vantage points. The model with equal detection probability was the best model (Δ AIC_c_ = 0), while the second model had Δ AIC_c_ > 2. The best model estimated a detection probability of 0.97 (SE = 0.04). This revealed a density estimate of 9.7 indiv./km^2^ (CV = 31.3%; 95% CI = 3.7–15.6) and an abundance of 519 individuals (95% CI = 201–837).

#### Small livestock

**Fig 2:**
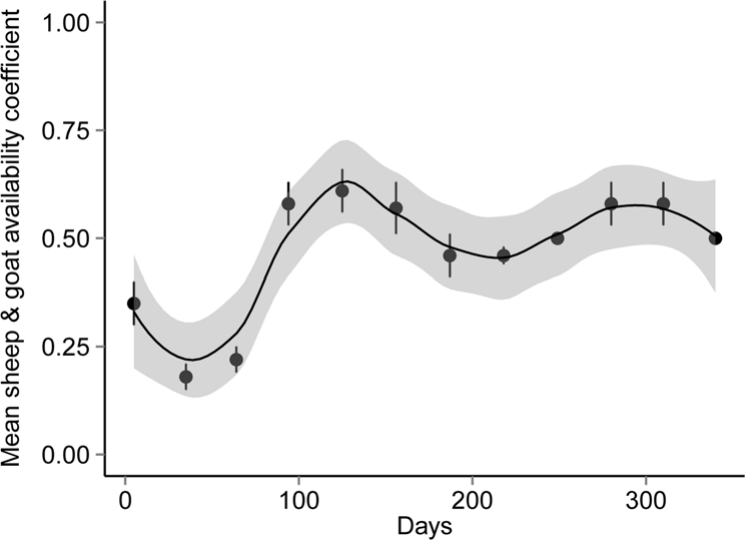
Temporal variation (with confidence intervals) in average monthly grazing of sheep and goats outside conflict villages of Golestan National Park.

From the interview surveys, small livestock depredation by leopard was reported in 96 cases in six villages < 2.5 km away from GNP (S2 Table), comprising 80.7% of all cases reported in the prior year (March 2012 to 2013). The total number of small livestock was reported as ~11250 individuals in the six conflict villages (S2 Table). Interviewees did not report any surplus killing by leopards. The annual average small livestock availability coefficient (*C*) for the six conflict villages ranged between 0.40 and 0.63 (Fig 2; S3 Table). In the village with the highest small livestock availability coefficient (*C* = 0.63), most of depredation cases were reported (41.7%). The total number of small livestock corrected for their availability was ~6392 individuals (S3 Table). This means that small livestock were by almost half (43.2%) less available than if we took the total stock of 11250 individuals as an indicator of availability. The average small livestock loss in conflict villages was around 1.7% ± SE 0.01 (0.2–6.0% per village) of the total population.

### Leopard prey preferences

**Fig 3:**
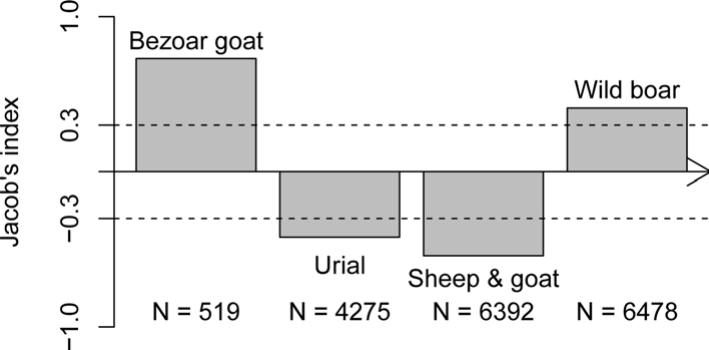
Persian leopard prey preference in Golestan National Park based on Jacob’s index (in the order of increasing abundance N). Jacob’s index >0.3 was considered as preferred and <-0.3 as avoided, with the index between these values indicating predation proportional to abundance [19].

**Fig 4:**
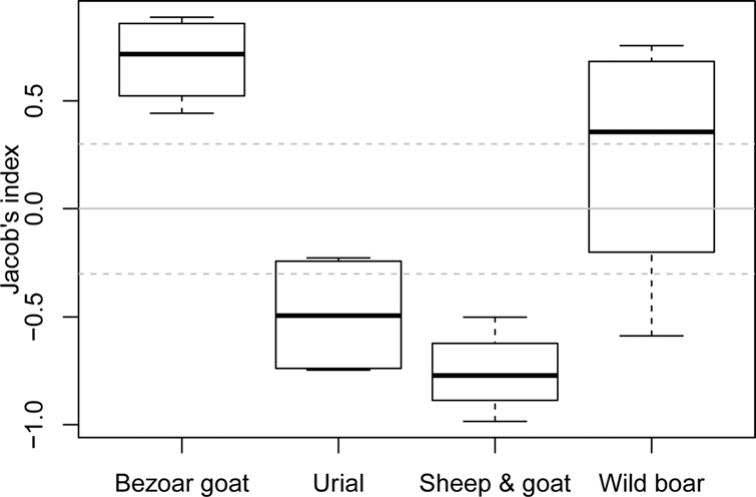
Results of sensitivity analysis of Persian leopard prey preference in Golestan National Park based on different combinations of the prey abundance 95% confidence intervals and corrected and uncorrected small livestock numbers.

In our study, wild boar was marginally preferred (I_j_ = 0.41; Table 2; Fig 3). However, bezoar goat was the most preferred species although it was the rarest (I_j_ = 0.73; Fig 3). Urial (I_j_ = -0.42; Table 2; Fig 3) and small livestock (I_j_ = -0.54; Table 2; Fig 3) were avoided. When small livestock were excluded from our study (as in most previous studies), bezoar goat was still preferred (I_j_ = 0.59) and urial avoided (I_j_ = -0.65). However, wild boar predation was estimated to be according to its abundance (I_j_ = 0.01). Moreover, when the absolute small livestock number (~11250) was used instead of the corrected one (~6392), leopards strongly avoided small livestock (I_j_ = -0.71). The results of the sensitivity analysis show that even using extreme variations in the prey abundance estimates, leopard preference for bezoar goat and avoidance of urial and small livestock remains unchanged. However, the role of wild boar in leopard diet under different prey abundance scenarios was different (Fig 4).

**Table 2:** The prey selectivity and numbers of prey individuals consumed by Persian leopard in Golestan National Park, Iran, in relation to abundance of the top four prey species.

Species	Corrected individuals consumed^a^ (%)	Abundance (95% CI^b^)	Abundance (%)	Jacob’s index
**Wild boar *Sus scrofa***	58.1	6478 (3050–9906)	36.7	0.41
**Bezoar goat *Capra aegagrus***	15.8	519 (201–838)	2.9	0.73
**Urial *Ovis vignei***	11.5	4275 (2117–8632)	24.2	-0.42
**Domestic goat *C*. *a*. *hircus* + domestic sheep *Ovis aries***	14.5	~6392	36.2	-0.54
**Total**	~100.0	17,664	100	-

^a^ based on *CF*_2;*i*_ = 3.094*exp* (−0.5((*ln*(*x*/16.370))/2.584)^2^) [34]

^b^ confidence interval

## Discussion

Our current understanding of the role of livestock in leopard diet is still limited. The most comprehensive review of leopard prey preference [16] ignores the role of livestock despite its presence in leopard diet in many parts of the world. Two recent studies [12–13], which considered livestock in their calculations, used only crude livestock numbers. Both these studies show that livestock is being avoided by leopards. In this study, we identified variation in sheep and goat availability to leopard depredation based on husbandry methods practiced across the conflict villages, which may affect the choice of this type of prey. When sheep and goat numbers are corrected for their availability outside villages, the leopard appears to still avoid them. Sheep and goats usually graze in large flocks and benefit from anti-predator protection, which possibly makes risks affiliated with this predation high [5, 11]. Preying on sheep and goats accompanied by shepherds and dogs may require a relatively longer time to ambush, capture and subdue, increasing the costs of hunting [5]. Moreover, livestock is only temporarily available within leopard habitats, what makes them a less reliable prey [14]. It suggests that leopards exhibit low preference for sheep and goats compared to other prey species in GNP, hinting that current husbandry methods can be effective in controlling depredation losses, but not in minimizing them [9, 11, 54]. We encourage the inclusion of livestock availability using such case-specific spatiotemporal corrections in assessing the role of livestock in felid diets and measuring the effectiveness of different husbandry practices in conflict sites.

Despite the avoidance of sheep and goats by leopards in GNP, depredation still occurs and causes considerable financial losses to local livelihoods. Therefore, current levels of conflict may arise from other factors that the results of this study may help conservation managers in better understanding them. Lack of sufficient prey is proposed as the major driver of big cat depredation on livestock [10]. The biomass of bezoar goat, wild boar and urial, which make 75% of total biomass consumed by leopards, in GNP is estimated as 717.09 kg/km^2^, which is above the minimum prey biomass threshold that drives sheep and goat depredation (544.57 ± 1.19 kg/km^2^) [10]. However, we found that local leopards avoided the urial, a steppe-dwelling species, despite its higher availability and appropriate body mass [19]. In other parts of the world, leopards also avoid open landscapes as a hunting habitat [17–18]. When excluding urial biomass, the prey biomass falls at the threshold of high sheep and goat predation (550.79 kg/km^2^). This may suggest that leopards in GNP are under pressure from insufficiency of natural preferred prey due to rampant ungulate poaching and are forced to take risky prey such as small livestock [19–20, 25]. The apparent avoidance of urial suggested by our analysis should be an important aspect in the conservation of Persian leopards throughout its range. This prey (along with mouflon *O*. *orientalis*) occurs in many habitats of Persian leopard and is usually considered as one of its main prey species. However, it is likely that the role of this species in leopard diet was overestimated in the past.

Sheep and goat body mass falls within the preferred prey range of leopard and this prey is docile [7, 19]. Although sheep and goats are kept on average 37–60% of a year within the studied villages, we have no reports of leopard attacks inside village pens. Therefore, it appears that penning has been an efficient tool to curb depredation, but herding practices and the use of guarding dogs during free grazing should be improved [52]. Incidents of leopard depredation on livestock may be related to the periods of lax herding (e.g. harvest season) or straggling livestock individuals [2, 4, 8]. The role of veterinary services in human-leopard conflict is also important in GNP [22]. Poor health condition may cause livestock to straggle and make them an easy prey for leopard, especially when they are unattended. The presence of conflict only in certain villages in our study may be related to vegetation cover, ruggedness of pastures [37] or occasionally straggling livestock [22]. Satisfaction with veterinary services in the conflict villages was 17% compared to 80% in no-conflict villages (S2 Table) [22]. Therefore, further investigations of livestock diseases in the area, as well as case-specific inspection of depredation site covariates are required to better understand conflict dynamics in the area. We also recorded cases of leopard attacks on sheep and goats in presence of shepherds and dogs. Such a readiness to take the risk of attacking livestock may be driven by individual characteristics such as body condition, experience and other behavioral factors [5]. A previous study [7] has shown that some carnivore individuals may display different patterns in daring livestock depredation, which may be linked to the individual’s sex and age. High depredation rate in some conflict villages (e.g. 6% loss of total livestock population in one village) may be due to behavior of certain culprit leopards (S2 Table). Future genetic analysis for the identification of individuals responsible for depredation of livestock may clarify this pattern.

Unlike earlier assumptions [16, 18] that leopards may use rocky outcrops only as refuges from larger predators and not for foraging, in GNP leopards prefer to hunt bezoar goats, which live in rocky areas. Leopards may have preferred bezoar goats because of suitability of rocky habitats for ambush hunting. However, the bezoar goat population in GNP suffers from an over 80% decline since the 1970’s and is extirpated by poachers from much of its former range [55]. We suppose that scarcity of bezoar goats is one of the main, if not the key factors contributing to leopard depredation on livestock in villages near GNP. Conservation actions to avert the bezoar goat population from further decline should become a top priority to reduce human-leopard conflict. In this study, wild boar represented most of prey biomass in leopard diet, but was only slightly preferred. Leopards avoid preying on members of the family Suidae throughout their range, possibly due to large body mass and aggressive behavior [12, 16, 19]. We cannot confirm whether high predation on wild boars in GNP is due to higher body mass of Persian leopards, a shift from bezoar goat toward a less preferred prey, or lack of competition with other large carnivores [19].

We acknowledge some limitations in our study design and data analyses, which might affect the interpretation of results. Our scat sampling was conducted over three years and the data on sheep and goat depredation by leopards and prey abundance were collected only in one or two of these years. However, we have no ground to surmise that leopard diet or prey abundances experienced any significant changes during this period. We also acknowledge the levels of uncertainty in our prey abundance data. However, this is of less importance as our sensitivity analysis showed that the main conclusions regarding preference or avoidance of prey are mostly valid under different abundance scenarios. In this study, we did not consider the spatial availability of wild prey. Wild prey is not equally distributed in the park, but leopard movements are not restricted to specific habitats and finding scats containing species not inhabiting the surrounding areas is frequent. Therefore, consideration of spatial variation in wild prey availability may not change our conclusions. There can be a potential bias from non-random camera placement in our wild boar abundance estimation because camera traps were set to capture leopards. Although leopard movement patterns may differ from those of wild boars, due to their predator-prey relationships, a correlation between the distribution and movements of these two species may exist. However, random encounter models yielded a very similar estimate of wild boar abundance (4890 indiv., 95% CI = 4188–5592) to line transects (4869 indiv., 95% CI = 2453–9664; S1 File) in GNP forests. Nevertheless, a potential bias arising from the difference in species daily movements in our and the reference study [43] remains unaccounted for. We also recognize the small sample size of our bezoar goat data, but the population estimate of this species in GNP is comparable with an earlier study, which used a different methodology (759 indiv., 95% CI = 583–935) [47].

Rearing sheep and goat in Iran and generally in Southwest Asia in presence of large carnivores has a long history and normally involves attendance of shepherds and dogs when herding outside villages. These practices may reduce the preference of domestic prey by leopards, but the situation can be different in other cases. Thus, prey preference studies may help conservationists in identification of preferred wild prey in felid diets and the estimation of prey availability, which may drive livestock depredation [10].

## Conclusions

We assessed livestock availability and identified domestic prey choice by leopards using spatiotemporal patterns of conflict distribution and husbandry methods. We conclude that leopards avoid sheep and goat depredation, which may suggest that in our case study, husbandry methods can be efficient in controlling the human-leopard conflict. Therefore, the cases of leopard depredation on livestock in our study area may be due to low abundance of preferred prey, (occasional) failures in herding practices, or characteristics of individual leopards. We suggest that future prey choice studies in conflict sites should pay attention to livestock availability in a way that incorporates the husbandry methods practiced.

## Supporting Information

S1 Fig Accumulation curve of prey species diversity in leopard scat samples. (PDF)

S1 File Line transect sampling data on wild boar in Golestan National Park. (XLSX)

S1 Table Top four prey species of leopard from scat sampling [29] and the pooled data with this study. (XLSX)

S2 Table Domestic sheep and goat numbers, reported killed numbers by leopard and satisfaction with veterinary services in the villages <2.5 km from Golestan National Park borders. (XLSX)

S3 Table Interview surveys with shepherds in conflict villages. (XLSX)
