# Supplementary material for: Assessing the Role of Livestock in Big Cat Prey Choice Using Spatiotemporal Availability Patterns
Source: PLoS One. 2016 Apr 11;11(4):e0153439. doi: 10.1371/journal.pone.0153439 (PMC4827856; doi:10.1371/journal.pone.0153439)
Supplement: S1 Fig — (PDF) [file pone.0153439.s001.pdf]

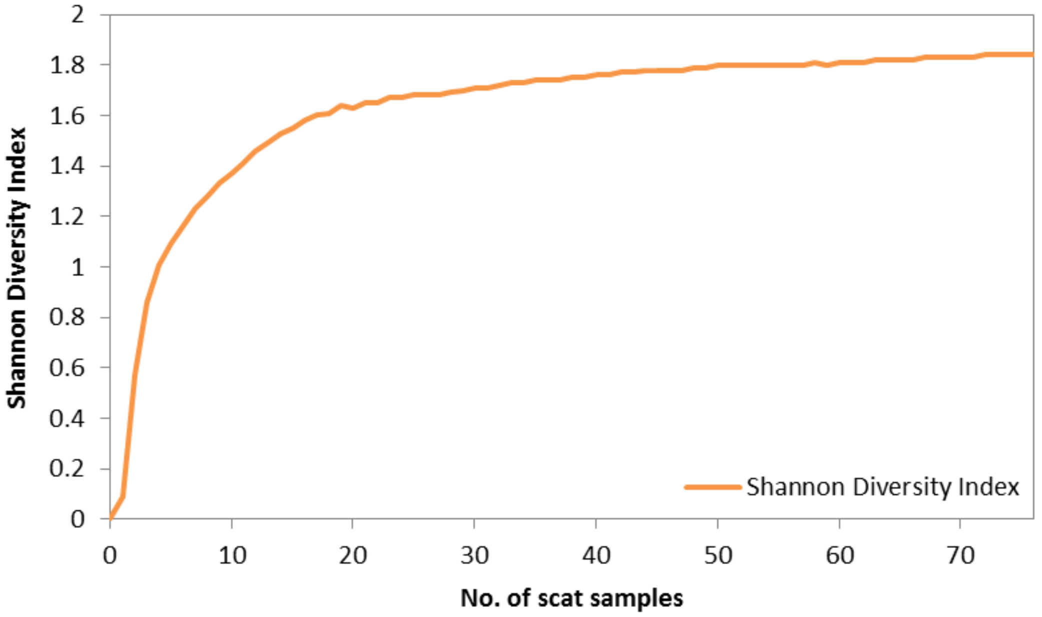

Shannon Diversity Index accumulation curve for the number of prey species in leopard diet in relation to scat numbers in Golestan National Park
